# Abemaciclib plus fulvestrant for the treatment of hormone receptor-positive/human epidermal growth factor receptor 2-negative breast cancer with cystic brain metastases: A case report and literature review

**DOI:** 10.3389/fonc.2022.984454

**Published:** 2022-11-30

**Authors:** Zhaohui Chu

**Affiliations:** Department of Oncology, Huashan Hospital, Fudan University, Shanghai, China

**Keywords:** breast cancer, cystic brain metastases, abemaciclib, fulvestrant, radiotherapy

## Abstract

Cystic brain metastases (CBM) in patients with breast cancer are rare. They have a worse prognosis than solid brain metastases, and they are less sensitive to radiotherapy. We report a case of hormone receptor-positive (HR+)/human epidermal growth factor receptor 2-negative (HER2−) metastatic breast cancer with CBM. The patient underwent treatment with docetaxel combined with capecitabine for 5 months, followed by anastrozole maintenance therapy for 10 months, and palbociclib combined with exemestane for 22 months. CBM emerged and bone metastases increased in number. A missense mutation in *PIK3CA* (exon 10, c.1633G>A [p.Glu545Lys]) was detected by whole-exome next-generation sequencing from peripheral blood samples. After whole-brain radiotherapy (40 Gy/20 fx) combined with 3 months of treatment with everolimus and fulvestrant, CBM demonstrated partial remission (PR), but extracranial bone metastases continued to increase in number. Thus, the patient underwent fourth-line treatment with abemaciclib (100 mg bid) combined with fulvestrant (500 mg). Three months later, CBM significantly demonstrated PR and extracranial bone metastases were stable. At present, the patient has above 9 months of progression-free survival time without obvious adverse effects. This is the first report of abemaciclib combined with fulvestrant in the treatment of CBM in a patient with HR+/HER2− breast cancer.

## Introduction

The incidence of cystic brain metastases(CBM) is very low, and the proportion of primary tumors that originate from breast cancer is second only to lung cancer (1). CBM may correlate with the greater malignancy and the more rapid growth of tumor, which more likely occurred in patients with lung cancer with genetic mutations, particularly epidermal growth factor receptor mutation or anaplastic lymphoma kinase positive non-small cell lung cancer. Compared with solid brain metastases (SBM), patients with CBM have much worse response to targeted therapy and shorter survival time (2). Similarly, for breast cancer, CBM was more aggressive than SBM, which was often seen in younger patients (3). Hormone receptor-positive (HR+)/human epidermal growth factor receptor 2-negative (HER2−) , triple negative and HER2 positive breast cancer accounts for 45.7% vs 55.7% , 31.4% vs 22.0% and 20.0% vs 18.8% of 35 patients with CBM compared with 255 patients with SBM. However, there has no obvious difference of tumor subtype in the presence between CBM and SBM (3). Three factors are significantly associated with an increased risk of CBM, including age of ≤40 years at the first breast cancer diagnosis, age of ≤45 years at the onset of brain metastases, and a poor histological grade. Compared with SBM, CBM are associated with a shorter median progression-free survival (PFS) time (4.2 vs 8.2 months) and a shorter median overall survival (OS) time (10.2 vs 17.0 months) (3). They are relatively insensitive to radiotherapy. CBM is more likely to recur than SBM when managed with stereotactic radiation (4). It is often larger than SBM. After stereotactic cyst aspiration and radiosurgery, the median intracranial PFS and OS for CBM were only 5.2 and 6.8 months (5). Therefore, effective drug therapies are needed to improve the prognosis. Abemaciclib has been approved for the treatment of HR+/HER2− metastatic breast cancer and has the advantage of passing through the blood–brain–barrier over other CDK4/6 inhibitors (6, 7). The present case report reported that in a patient with HR+/HER2− breast cancer CBM, abemaciclib and endocrine therapy as a fourth-line treatment have reduced CBM and controlled extracranial metastases even after progression on palbociclib.

## Case report

A 49-year-old postmenopausal female presented with two masses in the left breast. She underwent a modified radical mastectomy for left breast cancer in March 2018. Postoperative pathology confirmed that the 3.5 × 3 × 2-cm mass in the outer upper quadrant and the 1 × 0.8 × 0.8-cm mass in the inner quadrant were non-specific invasive breast cancer (grade II, left axillary lymph node 16/17[+]). Immunohistochemistry showed the following results: ER (90% strong positive), PR (75% strong positive), ki67 (35% positive), and HER2 (1+). One month after surgery, positron emission tomography/computed tomography showed multiple lung metastases, left hilar lymph node metastases, mediastinal lymph node metastases, left internal mammary lymph node metastases, and multiple bone metastases (C6 and L5 vertebral bodies, right third rib, left fourth rib, and right pubic bone). The patient was diagnosed with HR+/HER2− metastatic breast cancer. The treatment and outcomes are shown in **Table 1**.

**Table 1:** Treatments and outcomes before and after cystic brain metastases in a patient with hormone receptor-positive/human epidermal growth factor receptor 2-negative metastatic breast cancer.

Treatment	Regimen	Efficacy	Start and end dates of treatment	PFS time (months)	PD details
First-line treatment	Docetaxel + capecitabine q3w × 6 cycles, followed by 10 months of anastrozole maintenance therapy	PR	2018.4.25– 2018.8.13	15	Metastatic lung, left hilar lymph node, mediastinal lymph node, and right hilar lymph node lesions were enlarged
Second-line treatment	Palbociclib + exemestane	PR	2019.7.25– 2021.5.17	22	Brain metastases emerged and bone metastases increased in number
Third-line treatment	Whole-brain radiotherapy (40 Gy/20 fx) + everolimus + fulvestrant	Intracranial PR	2021.5.25– 2021.6.21	3.5	Bone metastases increased in number
	Everolimus + fulvestrant		2021.6.22– 2021.9.8		
Fourth-line treatment	Abemaciclib + fulvestrant	Intracranial PR, extracranial SD	2021.9.14– Present (2022.6.25 the last assessment)	>9	

Docetaxel, 75 mg/m^2^ ivgtt on day 1 Q3W; capecitabine, 1 g/m^2^ bid po on days 1–14 Q3W; anastrozole, 1 mg qd po; palbociclib, 100 mg on days 1–21 po Q4W; exemestane, 25 mg qd po; everolimus, 10 mg qd po; fulvestrant, 500 mg Q4W im (on days 1, 15, and 28 im the first three times); abemaciclib, 100 mg bid po; PFS, progression-free survival; PD, progressive disease; PR, partial remission; SD, stable disease.

**Figure 1:**
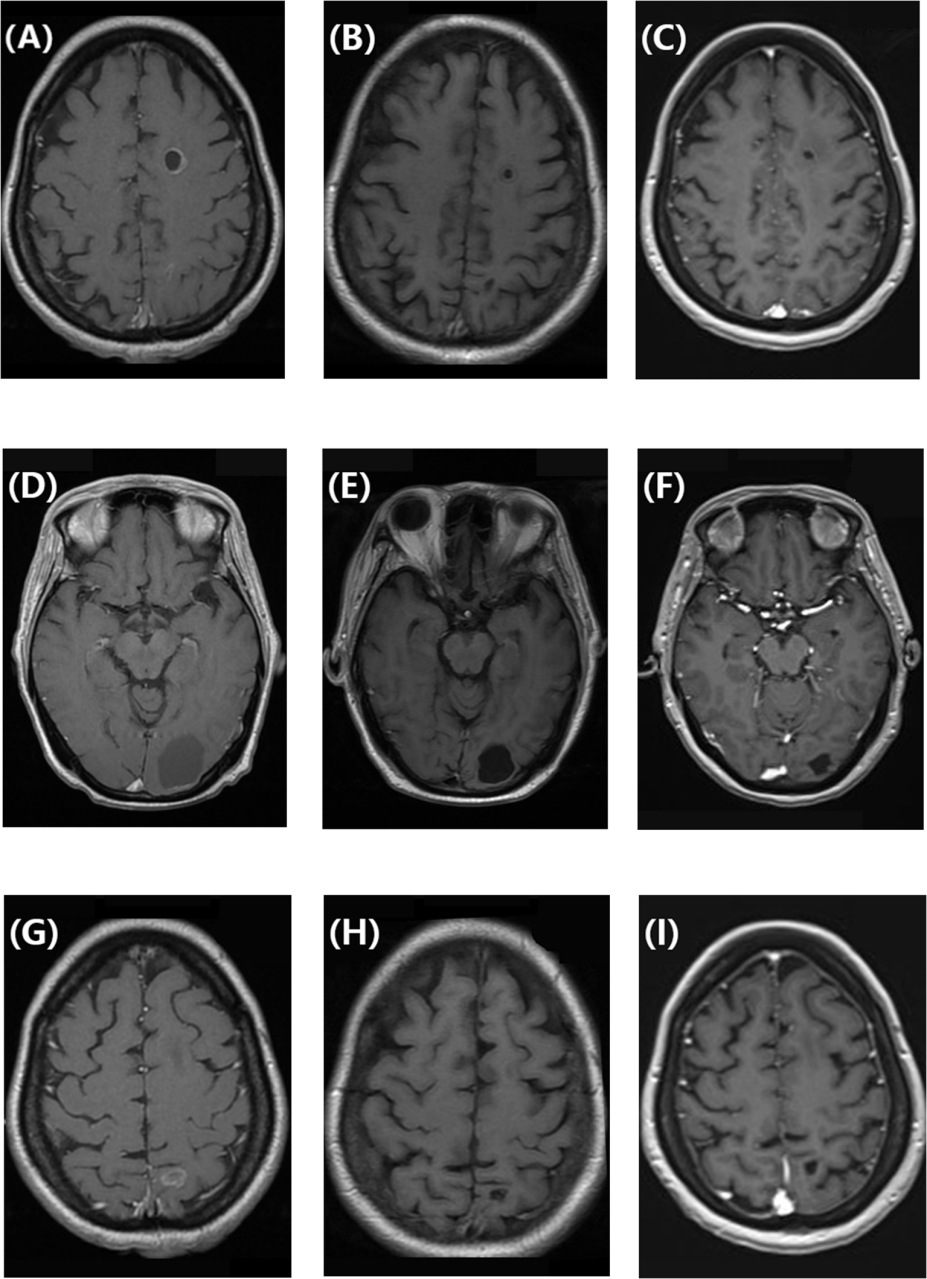
**(A, D, G)** Enhanced T1-weighted brain magnetic resonance image showing cystic brain metastases in a patient with hormone receptor-positive/human epidermal growth factor receptor 2-negative breast cancer before treatment with abemaciclib and fulvestrant. **(B, E, H)** Enhanced T1-weighted magnetic resonance image showing that the cystic brain metastases decreased in size after 3 months of treatment with abemaciclib and fulvestrant. **(C, F, I)** Enhanced T1-weighted magnetic resonance image showing that the cystic brain metastases remained in partial remission after 9 months of treatment with abemaciclib and fulvestrant.

This patient without visceral crisis accepted chemotherapy as first-line treatment because CDK4/6 inhibitors were unavailable in China at that time. Her visceral metastases were symptomatic and tumor progress was fast. According to the breast cancer guideline of Chinese Society of Clinical Oncology in 2018, we assessed that endocrine therapy alone may not be able to control tumors. So we recommended chemotherapy first and then endocrine therapy as maintenance therapy, and the patient acquired 15 months of PFS. After 22 months of second-line treatment with palbociclib combined with exemestane, the patient developed brain metastases for the first time. All of the five lesions were cystic, with a maximum diameter of 4 cm. They were located in left frontal lobe, left occipital lobe, and bilateral parietal lobe respectively. The number of bone metastases increased, and both extracranial and intracranial progressive diseases were observed. A missense mutation in *PIK3CA* (exon 10, c.1633G>A [p.Glu545Lys]) was detected by whole-exome next-generation sequencing from peripheral blood samples, and the mutation rate was 12.05%. After whole-brain radiotherapy (40 Gy/20 fx) combined with 3 months of treatment with everolimus and fulvestrant, the CBM partially disappeared and decreased in size, but the number of bone metastases increased. In 2021, a retrospective study reported that 87 patients receiving abemaciclib therapy after prior progression on palbociclib had a median PFS time of 5.3 months (8). So that fourth-line treatment was continued to retain fulvestrant, and everolimus was changed to abemaciclib. She took 150mg bid in the first two weeks and then reduced it to 100 mg bid due to intolerable diarrhea. After 3 months, the size of CBM significantly decreased (**Figure 1**), and extracranial bone metastases were stable. Until present, the patient has above 9 months of PFS without obvious adverse effects such as diarrhea, bone marrow suppression, or liver and kidney dysfunction. The patient had a good quality of life.

## Discussion

We presented a case of HR+/HER2− breast cancer with CBM. The following clinicopathological characteristics were observed: (1) A missense mutation in *PIK3CA* (exon 10, c.1633G>A [p.Glu545Lys]) in circulating tumor DNA from peripheral blood; (2) The CBM lesions were decreasing while the patient was on fulvestrant and everolimus. It continued to decrease after therapy was changed to fulvestrant and abemaciclib. (3) Treatment with abemaciclib combined with fulvestrant as salvage therapy was effective after successive treatment with palbociclib and everolimus.

CBM is more common in patients with primary lung cancer than in patients with breast cancer. They have been noted in patients with lung cancer with *ALK* rearrangement and *RET* fusion gene mutations (9, 10). There has been reported that cystic necrotic brain metastases were seen more often in triple-negative breast cancer (11), which maybe is owing to the rapid growth of tumor cells. However, breast cancer with CBM resulting from a gene mutation or after progression on palbociclib is rarely seen. In the present case, the missense mutation in *PIK3CA* (exon 10, c.1633G>A [p.Glu545Lys]) was somatic. This mutation is related to the occurrence of breast cancer (12), but it has not yet been reported to correlate with brain metastases. The mutation was identified at the time of the appearance of CBM after treatment with palbociclib combined with exemestane. There is no data about whole-exome next-generation sequencing before palbociclib treatment as a contrast. So that the mutation maybe is not a drug-resistant gene. Further investigation will be required to identify if this observation was coincidental or if there is an association between this mutation and CBM.

The patient in the present case had multiple CBM, with a maximum diameter of >3 cm. Therefore, whole-brain radiotherapy was preferred. However, considering the limited efficacy of radiotherapy alone for CBM, drug treatments were also used. The *PI3KCA* mutation observed in this patient is not considered a hot-spot mutation according to previous research. Instead, the *PIK3CA* E542K, E545X, and H1047X mutations are classified as hot-spot mutations owing to their longer PFS time with alpelisib plus fulvestrant treatment (PI3Kα inhibitor) compared with fulvestrant alone in the SOLAR-1 study (13). These hot-spot mutations also correlated with a longer PFS time when applying alpelisib after the progression of an aromatase inhibitor with a CDK4/6 inhibitor in the BYLieve study (14). However, alpelisib was not available in China. Instead, we used everolimus (an mTOR inhibitor) to target the downstream PI3K/Akt/mTOR signal pathway. Unfortunately, the results were not satisfactory; the PFS time was only 3.5 months, which is not comparable to the PFS time of 8.8 months observed with everolimus plus exemestane treatment in patients with the *PIK3CA* H1047 mutation (15). Thus, the reduction in the size of CBM is mainly considered to be the effect of whole-brain radiotherapy.

Given that CyberKnife stereotactic radiotherapy demonstrates a PFS time of only 3 months in patients with CBM (1), effective drug treatments are still needed to consolidate the effect of radiotherapy. At that time, a multicenter retrospective study reported that patients undergoing treatment with abemaciclib with or without endocrine therapy achieved a median PFS time of 5.3 months and a median OS time of 17.2 months after prior progression on palbociclib (8). However, the efficacy of using another CDK4/6 inhibitor (abemaciclib) after progression on CDK4/6 inhibitor (palbociclib) for brain metastases especially CBM has rarely been reported.

Preclinical studies have shown that abemaciclib inhibits the growth of ependymoma in the xenograft tumor model (16). Moreover, a phase I clinical trial of abemaciclib showed that its concentration in cerebrospinal fluid was similar to that in plasma (17). A phase 2 clinical trial has reported that (18), three patients with HR+/HER2− brain metastases resulting from breast cancer underwent resection of brain lesions after 5–14 days of abemaciclib treatment (200 mg bid), and the concentration of abemaciclib in brain metastatic tissue was similar to that in cerebrospinal fluid and plasma. In cohort A including 58 patients with HR+/HER2−breast cancer brain metastases, 46.6% underwent whole-brain radiotherapy, 34.5% underwent stereotactic radiotherapy, 6.9% underwent surgical resection for brain metastases, 70.7% underwent endocrine therapy, and 27.6% underwent targeted therapy. After treatment by abemaciclib (200 mg bid) with or without endocrine therapy, the size of the brain metastases reduced in 38% of patients, and 5.2% of patients achieved PR (18). In this case report, treatment with abemaciclib and fulvestrant significantly reduced the size of CBM after 3 months and controlled extracranial and intracranial metastases for above 9 months, which demonstrates the advantage of abemaciclib in penetrating the blood–brain–barrier.

The MONARCH 3 clinical trial (19) showed that abemaciclib combined with fulvestrant as second-line therapy for patients with HR+/HER2− breast cancer with extracranial metastases has significant survival benefits, with a median PFS time of 16.4 months and a median OS time of 46.7 months. Abemaciclib as a median sixth-line treatment after prior progression on palbociclib achieved a median PFS time of 5.3 months (8). In the MAINTAIN trial, ribociclib plus endocrine therapy after progression on endocrine therapy plus CDK4/6 inhibition (palbociclib in 87% cases) showed a median PFS of 5.29 months (20). In the present case, the patient experienced progressive disease after undergoing chemotherapy, aromatase inhibition, and targeted therapy with palbociclib and everolimus. Abemaciclib combined with fulvestrant as the fourth-line therapy showed benefit in terms of a PFS time of above 9 months. Therefore, the benefit of abemaciclib combined with fulvestrant might apply to all lines of treatment, and the survival benefit may be greater the earlier it is used.

In conclusion, we reported a case in which treatment with abemaciclib combined with fulvestrant as the fourth-line salvage therapy for metastatic breast cancer was effective in reducing CBM. In the future, the clinical research about the treatment of CBM in HR+/HER2− breast cancer patients should be conducted.

## Data availability statement

The original contributions presented in the study are included in the article/supplementary material. Further inquiries can be directed to the corresponding author.

## Author contributions

ZC is responsible for conception and ideas of this study, collecting the data, interpreting the data and writing paper for this study. She has also did the submission of this manuscript.

## Funding

This study was funded by grants from Clinical Research Fund of Chinese Society of Clinical Oncology (Y-HR2018-026).

## Conflict of interest

The author declares that the research was conducted in the absence of any commercial or financial relationships that could be construed as a potential conflict of interest.

## Publisher’s note

All claims expressed in this article are solely those of the authors and do not necessarily represent those of their affiliated organizations, or those of the publisher, the editors and the reviewers. Any product that may be evaluated in this article, or claim that may be made by its manufacturer, is not guaranteed or endorsed by the publisher.
